# Supplementary material for: MEME-LaB: motif analysis in clusters
Source: Bioinformatics. 2013 May 14;29(13):1696–7. doi: 10.1093/bioinformatics/btt248 (PMC3694638; doi:10.1093/bioinformatics/btt248)
Supplement: Supplementary Data [file supp_btt248_Supplementary_Data.zip › Supplementary_Data/Results_Files/which_gene_ids/cluster20_motif1.html]

Cluster 20 Motif 1


Cluster 20 Motif 1 is found in the following promoters

| Gene ID | Strand | Position\* |
| --- | --- | --- |

|  |  |  |
| --- | --- | --- |
| AT1G01740 | - | 417/500 |
| AT1G02780 | - | 1/500 |
| AT1G02870 | - | 139/500 |
| AT1G03860 | + | 379/500 |
| AT1G04510 | + | 323/500 |
| AT1G04520 | - | 60/500 |
| AT1G04870 | - | 273/500 |
| AT1G04985 | + | 481/500 |
| AT1G05180 | - | 304/500 |
| AT1G05210 | - | 478/500 |
| AT1G05790 | - | 295/500 |
| AT1G08125 | + | 408/500 |
| AT1G08360 | + | 204/500 |
| AT1G09590 | + | 71/500 |
| AT1G10320 | + | 161/500 |
| AT1G10450 | + | 307/500 |
| AT1G10490 | + | 387/500 |
| AT1G10650 | + | 450/500 |
| AT1G10900 | - | 327/500 |
| AT1G12000 | - | 60/500 |
| AT1G13120 | - | 203/500 |
| AT1G15420 | - | 119/500 |
| AT1G16690 | + | 71/500 |
| AT1G16740 | - | 163/500 |
| AT1G17280 | - | 279/500 |
| AT1G21200 | - | 316/500 |
| AT1G26850 | + | 52/500 |
| AT1G28350 | - | 480/500 |
| AT1G28540 | + | 300/500 |
| AT1G29250 | - | 308/500 |
| AT1G29850 | - | 200/500 |
| AT1G30010 | - | 3/500 |
| AT1G30460 | - | 110/500 |
| AT1G30580 | + | 139/500 |
| AT1G31870 | + | 349/500 |
| AT1G31910 | - | 334/500 |
| AT1G32130 | - | 40/500 |
| AT1G32150 | - | 199/500 |
| AT1G32330 | + | 307/500 |
| AT1G34130 | - | 462/500 |
| AT1G34220 | - | 64/500 |
| AT1G43710 | - | 93/500 |
| AT1G47340 | + | 471/500 |
| AT1G48460 | + | 392/500 |
| AT1G48635 | - | 371/500 |
| AT1G49400 | + | 199/500 |
| AT1G50590 | + | 44/500 |
| AT1G51700 | + | 446/500 |
| AT1G52280 | - | 188/500 |
| AT1G52730 | + | 327/500 |
| AT1G53500 | + | 476/500 |
| AT1G53710 | + | 283/500 |
| AT1G54160 | - | 120/500 |
| AT1G55460 | - | 78/500 |
| AT1G56290 | + | 30/500 |
| AT1G56340 | + | 236/500 |
| AT1G58110 | + | 272/500 |
| AT1G58350 | + | 458/500 |
| AT1G60070 | + | 408/500 |
| AT1G60220 | - | 36/500 |
| AT1G61150 | - | 484/500 |
| AT1G61210 | + | 431/500 |
| AT1G61770 | + | 453/500 |
| AT1G64140 | - | 21/500 |
| AT1G67070 | - | 390/500 |
| AT1G68580 | + | 161/500 |
| AT1G69690 | + | 458/500 |
| AT1G71070 | - | 485/500 |
| AT1G71840 | + | 124/500 |
| AT1G72740 | + | 486/500 |
| AT1G74050 | + | 426/500 |
| AT1G76320 | + | 182/500 |
| AT1G76810 | + | 58/500 |
| AT1G78200 | + | 168/500 |
| AT1G78690 | + | 51/500 |
| AT1G80860 | + | 272/500 |
| AT2G02450 | - | 340/500 |
| AT2G03270 | + | 333/500 |
| AT2G04340 | + | 276/500 |
| AT2G05230 | + | 277/500 |
| AT2G06510 | + | 82/500 |
| AT2G12550 | + | 147/500 |
| AT2G14260 | + | 354/500 |
| AT2G15430 | + | 313/500 |
| AT2G16430 | - | 58/500 |
| AT2G16770 | + | 330/500 |
| AT2G17360 | - | 375/500 |
| AT2G17410 | + | 373/500 |
| AT2G18040 | - | 20/500 |
| AT2G19310 | + | 345/500 |
| AT2G25720 | + | 294/500 |
| AT2G26920 | + | 412/500 |
| AT2G27030 | - | 457/500 |
| AT2G27860 | + | 209/500 |
| AT2G27950 | + | 375/500 |
| AT2G27990 | + | 454/500 |
| AT2G28540 | + | 386/500 |
| AT2G29200 | + | 412/500 |
| AT2G29210 | + | 213/500 |
| AT2G30060 | + | 368/500 |
| AT2G30120 | - | 219/500 |
| AT2G30880 | + | 307/500 |
| AT2G31140 | + | 32/500 |
| AT2G31740 | + | 274/500 |
| AT2G32680 | - | 121/500 |
| AT2G32850 | - | 107/500 |
| AT2G32910 | - | 212/500 |
| AT2G32920 | - | 120/500 |
| AT2G33730 | + | 402/500 |
| AT2G34730 | + | 482/500 |
| AT2G34840 | - | 161/500 |
| AT2G35330 | - | 479/500 |
| AT2G36620 | + | 44/500 |
| AT2G38000 | - | 489/500 |
| AT2G39260 | + | 217/500 |
| AT2G39340 | - | 334/500 |
| AT2G39390 | + | 214/500 |
| AT2G40060 | + | 160/500 |
| AT2G40270 | + | 364/500 |
| AT2G41430 | + | 363/500 |
| AT2G41650 | + | 358/500 |
| AT2G44120 | - | 125/500 |
| AT2G45260 | + | 1/500 |
| AT2G45790 | - | 193/500 |
| AT2G46250 | + | 368/500 |
| AT2G47250 | - | 210/500 |
| AT2G47320 | + | 407/500 |
| AT2G47410 | + | 200/500 |
| AT2G47580 | + | 100/500 |
| AT2G47630 | - | 270/500 |
| AT3G01460 | + | 292/500 |
| AT3G02320 | - | 488/500 |
| AT3G02510 | + | 87/500 |
| AT3G02530 | + | 266/500 |
| AT3G03420 | + | 197/500 |
| AT3G03940 | - | 72/500 |
| AT3G03950 | + | 360/500 |
| AT3G04470 | + | 43/500 |
| AT3G05420 | + | 214/500 |
| AT3G06010 | - | 188/500 |
| AT3G06450 | + | 1/500 |
| AT3G10540 | + | 482/500 |
| AT3G12360 | - | 468/500 |
| AT3G13300 | + | 476/500 |
| AT3G13460 | + | 468/500 |
| AT3G13870 | - | 223/500 |
| AT3G14600 | - | 160/500 |
| AT3G15520 | + | 123/500 |
| AT3G16650 | - | 381/500 |
| AT3G17880 | - | 10/500 |
| AT3G18370 | + | 26/500 |
| AT3G18760 | + | 247/500 |
| AT3G19540 | - | 14/500 |
| AT3G19760 | - | 273/500 |
| AT3G19790 | + | 346/500 |
| AT3G20330 | + | 451/500 |
| AT3G20430 | - | 352/500 |
| AT3G20770 | - | 135/500 |
| AT3G20920 | - | 454/500 |
| AT3G21280 | - | 427/500 |
| AT3G23810 | + | 159/500 |
| AT3G24090 | - | 21/500 |
| AT3G24570 | + | 176/500 |
| AT3G26600 | + | 470/500 |
| AT3G26850 | + | 72/500 |
| AT3G27090 | + | 145/500 |
| AT3G27520 | - | 131/500 |
| AT3G42860 | + | 367/500 |
| AT3G44850 | + | 147/500 |
| AT3G47120 | + | 332/500 |
| AT3G48960 | - | 345/500 |
| AT3G50340 | + | 292/500 |
| AT3G52040 | - | 487/500 |
| AT3G52240 | - | 341/500 |
| AT3G52260 | + | 440/500 |
| AT3G52390 | + | 480/500 |
| AT3G53630 | - | 317/500 |
| AT3G53920 | + | 471/500 |
| AT3G54760 | + | 341/500 |
| AT3G55830 | + | 213/500 |
| AT3G55840 | - | 42/500 |
| AT3G57000 | - | 396/500 |
| AT3G57640 | + | 77/500 |
| AT3G58680 | + | 242/500 |
| AT3G60030 | - | 171/500 |
| AT3G61140 | - | 132/500 |
| AT3G61415 | - | 435/500 |
| AT3G61800 | - | 417/500 |
| AT3G62330 | - | 108/500 |
| AT4G00026 | + | 335/500 |
| AT4G00752 | - | 286/500 |
| AT4G01120 | - | 464/500 |
| AT4G01590 | + | 209/500 |
| AT4G01880 | + | 138/500 |
| AT4G01990 | + | 193/500 |
| AT4G02010 | + | 444/500 |
| AT4G02150 | + | 357/500 |
| AT4G03480 | + | 198/500 |
| AT4G08940 | + | 256/500 |
| AT4G10320 | + | 425/500 |
| AT4G10480 | + | 238/500 |
| AT4G13170 | + | 16/500 |
| AT4G14455 | - | 466/500 |
| AT4G14920 | - | 150/500 |
| AT4G16143 | + | 382/500 |
| AT4G18040 | + | 443/500 |
| AT4G18260 | + | 222/500 |
| AT4G19600 | - | 459/500 |
| AT4G21090 | - | 248/500 |
| AT4G22000 | + | 51/500 |
| AT4G22285 | - | 182/500 |
| AT4G22350 | + | 40/500 |
| AT4G22990 | - | 179/500 |
| AT4G23620 | - | 187/500 |
| AT4G23930 | + | 129/500 |
| AT4G26090 | - | 100/500 |
| AT4G26610 | - | 399/500 |
| AT4G28200 | + | 460/500 |
| AT4G28910 | - | 26/500 |
| AT4G31770 | - | 473/500 |
| AT4G31920 | - | 436/500 |
| AT4G32560 | - | 179/500 |
| AT4G35320 | - | 24/500 |
| AT4G35570 | + | 51/500 |
| AT4G35785 | - | 225/500 |
| AT4G36130 | + | 72/500 |
| AT4G36780 | + | 287/500 |
| AT4G37090 | + | 381/500 |
| AT4G37120 | + | 126/500 |
| AT4G38100 | + | 336/500 |
| AT4G38930 | - | 166/500 |
| AT4G39040 | + | 72/500 |
| AT4G39050 | + | 483/500 |
| AT4G39160 | + | 103/500 |
| AT4G39180 | - | 488/500 |
| AT4G39690 | + | 168/500 |
| AT4G39990 | - | 209/500 |
| AT5G03415 | + | 157/500 |
| AT5G03740 | - | 145/500 |
| AT5G06960 | - | 175/500 |
| AT5G07140 | + | 333/500 |
| AT5G07840 | - | 1/500 |
| AT5G08080 | - | 266/500 |
| AT5G08320 | - | 39/500 |
| AT5G08415 | - | 375/500 |
| AT5G11560 | + | 41/500 |
| AT5G11760 | + | 384/500 |
| AT5G13020 | + | 384/500 |
| AT5G14440 | + | 72/500 |
| AT5G15080 | + | 259/500 |
| AT5G15570 | - | 395/500 |
| AT5G17240 | + | 485/500 |
| AT5G17930 | + | 361/500 |
| AT5G19900 | + | 186/500 |
| AT5G20160 | + | 365/500 |
| AT5G20300 | + | 191/500 |
| AT5G20660 | + | 446/500 |
| AT5G22950 | - | 404/500 |
| AT5G23250 | + | 400/500 |
| AT5G23590 | + | 352/500 |
| AT5G23610 | + | 123/500 |
| AT5G26770 | - | 254/500 |
| AT5G27470 | - | 485/500 |
| AT5G27540 | + | 458/500 |
| AT5G29000 | - | 193/500 |
| AT5G35570 | - | 315/500 |
| AT5G35910 | - | 121/500 |
| AT5G38600 | + | 355/500 |
| AT5G41190 | + | 19/500 |
| AT5G41410 | + | 87/500 |
| AT5G42320 | + | 329/500 |
| AT5G42360 | - | 420/500 |
| AT5G42540 | - | 230/500 |
| AT5G43810 | + | 119/500 |
| AT5G44100 | - | 193/500 |
| AT5G45420 | + | 142/500 |
| AT5G45510 | + | 104/500 |
| AT5G45600 | - | 318/500 |
| AT5G48120 | - | 175/500 |
| AT5G49510 | - | 193/500 |
| AT5G49530 | + | 408/500 |
| AT5G50320 | + | 399/500 |
| AT5G50430 | - | 33/500 |
| AT5G50580 | - | 70/500 |
| AT5G50970 | + | 437/500 |
| AT5G52200 | + | 292/500 |
| AT5G53000 | + | 245/500 |
| AT5G53430 | + | 327/500 |
| AT5G53760 | - | 3/500 |
| AT5G56160 | + | 348/500 |
| AT5G56950 | + | 464/500 |
| AT5G57280 | + | 284/500 |
| AT5G60640 | + | 92/500 |
| AT5G60670 | + | 472/500 |
| AT5G61450 | + | 453/500 |
| AT5G62050 | + | 307/500 |
| AT5G63050 | + | 410/500 |
| AT5G64150 | - | 169/500 |
| AT5G64816 | - | 299/500 |
| AT5G66860 | + | 379/500 |
| AT5G67440 | - | 24/500 |

**\*** Overlapping sequences are merged to avoid redundancy. As a result some sequences in the final set may be longer than the specified maximum.

Distribution

+

-

Position values represent number of bases from the 5 prime end of the sequence provided to MEME and the length of that sequence. It is these figures expressed as proportions that are used for statistical analysis.

Probability value of comparison of these values to a linear distribution, p = 0.11

The motif is found 176 times on the + stand and 125 times on the - strand.

Probability value that it is evenly distributed between the two strands, p = 0.0019

Move the mouse pointer over the gene ids to highlight the corresponding marker
